# Investigating effects of parasite infection on body condition of the Kafue lechwe (*Kobus leche kafuensis*) in the Kafue basin

**DOI:** 10.1186/1756-0500-3-346

**Published:** 2010-12-23

**Authors:** Musso Munyeme, Hetron M Munang'andu, John B Muma, Andrew M Nambota, Demelash Biffa, Victor M Siamudaala

**Affiliations:** 1 Department of Disease Control, School of Veterinary Medicine, University of Zambia, P.O. Box 32379 Lusaka, Zambia; 2 Norwegian School of Veterinary Sciences, Department of Basic Sciences and Aquatic Medicine, Section of Aquatic Medicine and Nutrition, Ullevalsveien 72, P.O. Box 8146 Dept, NO-0033 Oslo, Norway; 3 Center for Epidemiology and Biostatistics, Norwegian School of Veterinary Science, P.O. Box 8146, N-0033, Oslo, Norway; 4 Zambia Wildlife Authority, Private bag 1, Chilanga, Zambia

## Abstract

**Background:**

The Kafue lechwe (*Kobus leche Kafuensis*), a medium-sized semi-aquatic antelope, is endemic to the Kafue basin of Zambia. The population of the Kafue lechwe has significantly dropped in the last decades leading to its subsequent inclusion on the red list of endangered species. In order to save the remaining population from extinction, it has become increasingly important that the impact of parasite infection and infestation on the Kafue lechwe is investigated.

**Findings:**

Endoparasites accounted for the majority of parasites observed from a study of 40 Kafue lechwe occurring in the the Kafue basin. *Amphistoma spp. *were present in all animals examined, while *Fasciola gigantica *had a prevalence rate of 0.525 (95% CI: 0.36 to 0.69) and species of *Schistosoma *0.3 (95% CI: 0.15 to 0.45). Among the ectoparasites, *Strobiloestrous vanzyli*, had a prevalence rate of 0.15 (95% CI: 0.03 to 0.27), while *Rhipicephalus appendiculatus *had a prevalence of 0.075 (3/40). Our findings indicate that body condition was not influenced by the parasitic infestation in Kafue lechwe. There was no association between sex and parasitic burden (odds ratio = 0.3, 95% CI: 0.8-1.3). However, an association between age and parasitic burden was observed as older animals above 15 years were more likely to get parasite infections than those aged between 1-5 years (odds ratio = 1.5, 95% CI: 1.1-2.4).

**Conclusion:**

Overall, there was no evidence that parasitic infections and infestations adversely affected the lechwe population on the Kafue basin. These findings indicate that ecto- and endo-parasite infestation might not play a significant role in reducing the Kafue lechwe population on the Kafue basin.

## Background

The Kafue lechwe antelope (*Kobus leche kafuensis*) is the predominant wildlife species of the Kafue basin. These antelopes are lek-breeders and share their habitat with other wild and domestic species. They are semi-aquatic and medium sized antelopes that live in large groups close to water bodies or marsh places [1]. The lechwe population on the Kafue basin has steadily declined from an estimated 80,000 in 1975 to 41,000 in 2001 [2,3] leading to its recent inclusion on the IUCN Red list of threatened species [4]. This has led the Zambia wildlife Authority (ZAWA) to embark on conservation programs aimed at saving the remaining population from going into extinct. In order to achieve this, it has become increasingly important to investigate all potential factors likely to contribute to the decline of the Kafue lechwe population.

The Kafue basin ecosystem harbors various species of ecto- and endo parasites that infest both wild and domestic animals [5-7]. Studies by Kampamba [8] and Kapungwe [3] indicate that ecological changes and animal diseases are the major factors reducing the Kafue lechwe population in the Kafue basin. Gallagher et al [9] estimated that parasitic diseases accounted for approximately 14% annual mortality of the lechwe population each year and formed a major factor limiting population growth. Previous studies have focused on identifying the species of parasites infesting the Kafue lechwe [9,10], did not investigate on the impact of these parasites. In cattle parasitic infestations have been associated with considerable weight loss, which in some cases have led to mortalities depending on the severity of the infection [11]. In evaluating animal health conditions, body-condition is used as a proxy for evaluating the health status of wildlife within a habitat and is thus a useful tool for assessing the impact of diseases on wild species [12]. In the present study, we investigated the effect of the ecto and endo-parasites found in the Kafue basin on body condition of free ranging Kafue lechwe, as a way of examining the role of parasite infestation on reducing the Kafue lechwe population.

## Materials and methods

### Study area and animals

**Figure 1:**
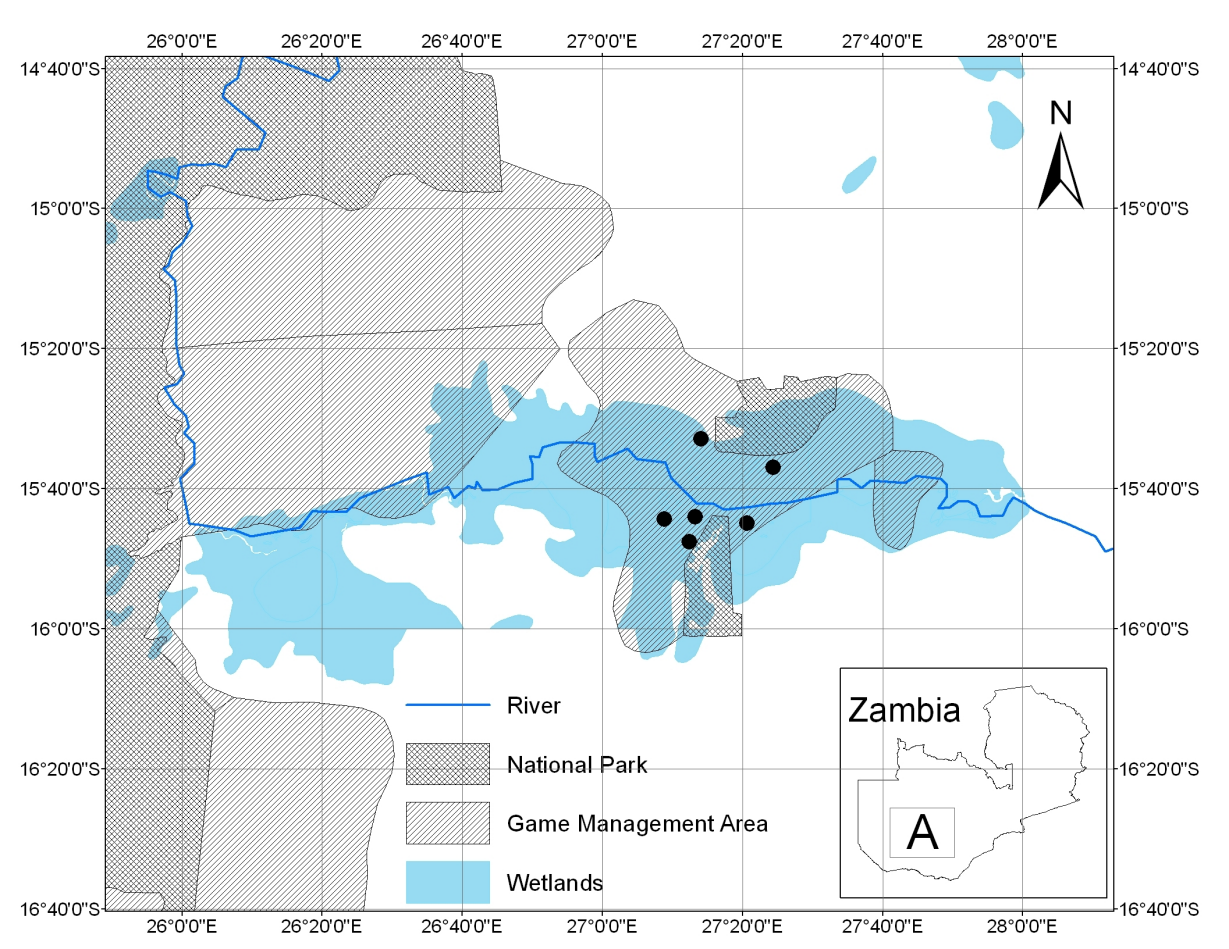
**Map of the Kafue basin showing the location of National Parks and Game Management Areas being partially covered by the wetlands**. Black dots show the sampling sites. The insert shows the map of Zambia with A showing the study area.

The study was conducted in the Kafue basin (Figure 1). The Kafue basin covers an area of about 6,000 km^2^, encompassing the Blue Lagoon National Park (420 km^2^), Lochinvar National Park (410 km^2^) and Kafue GMA (5,175 km^2^). This area supports grazing for cattle in the dry season resulting for active interaction between livestock and wildlife. Samples were obtained from six Kafue lechwe herds occurring within game management areas (GMA) (Figure 1). GMAs are ecological buffer zones between communal lands and National Parks (NP) allowing for the co-existence of livestock [13,14].

Given the endangered status of the Kafue lechwe [4], only 40 animals (20 females and 20 males) were sacrificed by ZAWA for the purpose of parasite investigations between October and December 2005. Ethical approval, under special license number 00113, was issued by the Zambia Wildlife Authority the sole custodians of wildlife in Zambia as stipulated in Section 37 of the Zambia Wildlife Act of the laws of the Republic of Zambia. Animals were aged using ring patterns on the horns as well as tooth development and wear [15,16]. Examination of carcasses was carried out as described by Gracey [17]. Body condition was graded using the kidney fat index (KFI) as described by Riney [18,19]. The amount of fat removed from the kidneys was weighed and divided by the total weight of the kidneys for each animal to get the index. Each carcass was allocated a score of 1 (poor: almost no fat); 2 (average: fair fat amount present); or 3 (good: plentiful fat, completely obscuring the kidneys).

### Sample collection and examination

All animals were eviscerated followed by visual inspection and digital palpation of all body organs. Contents of the thoracic cavity were examined separately. Each component of the digestive system was processed separately with the contents from each segment being placed in separate bottles for laboratory examination. *Schistosoma matteii *in the mesenteries were collected using forceps and counted at the sampling sites. The liver was incised and examined for the presence of flukes. Visceral contents from each organ were emptied into a bucket and the mucosa washed thoroughly in water with firm digital pressure.

Quantification of the parasites was achieved by sieving and sedimentation following the method described by Taira *et al *[20], while egg counts of *Schistosoma matteii *were examined as described by Lawrence [21]. Cysts were excised from the heart muscles and evaginated from cyst membranes using methods described by Edgar [22] and Gonzalez et al [23]. They were later identified as cysticercus of *Taenia saginata *as described by Okafor [24] and Opara et al [25].

### Statistical analysis

All observations were recorded in Microsoft Excel and later transferred to STATA™ V/SE 10 (Stata Corp, http://www.stata.com) for analysis. Logistic regression was used to determine the associations between body condition (based on the KFI) and parasite infection while the ROC test was used to determine the reliability of the model. Pearson's chi-square was used to determine the correlation of concurrent infections between different parasites.

## Results

### Descriptive analysis

Five genera of endo-parasites and two species of ecto-parasites were identified (Table 1). The endo-parasites comprised of *Schistosoma mattei *isolated from the mesenteric veins, *Fasciola gigantica *from the liver and species of *Amphistoma *from the abomasum and rumen. Species of *Setaria *were isolated from the peritoneal cavity in the abdomen, while cysts of cysticercus of *Taenia saginata *were collected from the muscles of the heart, tongue and inter-coastal region. Ectoparasites included oestrid larvae (*Strobiloestrous vanzyli*) isolated from the subcutis of the back and dorsal aspect (Table 2) and *Rhipicephalus appendiculatus *obtained from different body parts of the skin surface (Table 2).

**Table 1:** Crude parasitic infection in lechwe antelopes across the two sampling areas (Lochinvar and Blue Lagoon) (n = 40), indicative of crude prevalence values per type of parasite (October to December, 2005)

Parasite	No. of Lechwes tested	Proportion of Positive findings(%)	95% Confidence Interval
*Amphistoma spp.*	40	100	-
*Schistosoma spp.*	40	30	[15.2-44.8]
*Cysticercus bovis*	40	15	[3.4-26.6]
*Fasciola gigantica*	40	52.5	[36.3-68.7]
*Strobiloestrous vanzyli*	40	15	[3.4-26.6]
Wireworms*	40	35	[19.5-50.4]
Ticks	40	17.5	[5.2-29.8]

*These worms were found in the free space of the peritoneum

**Table 2:** Factors assessed in relation to parasite infestation in the Kafue lechwe antelopes (n = 40), showing proportions of distribution according to various levels (October to December, 2005).

Variable	Level	% proportion	95% CI
Sex	Females	47.5	[31.3-63.7]
	Males	52.5	[36.3-68.7]

Age	1-5 years	5	[0-12]
	5-10 years	60	[44.1-75.9]
	10-15 years	27.5	[13-41.9]
	>15 years	7.5	[0-16]

Body Condition Score	Good	75	[60.9-89]
	Fair	12.5	[1.8-23.2]
	Poor	12.5	[1.8-23.2]

*Fasciola gigantica *was the second most prevalent endo-parasite at a proportion of 52.5% (21/40) after species of *Amphistosoma *found in virtually all animals screened. Concurrent infections of *Amphistosoma *and *Schistosoma mattei *accounted for 17.5% (7/40), species of *Amphistosoma *and *Fasciola gigantica *20% (8/40), *Schistosoma mattei *and *Fasciola gigantica *17.5% (8/40), while a combination of all trematodes (trematodes *Amphistosoma species, Fasciola gigantica *and *Schistosoma mattei*) accounted for 12.5% (5/40). Co-infections of all trematodes and nematodes (*Setaria *spp.) accounted for 5% (2/40), while a combination of trematodes, nematodes and cestodes (cysts) accounted for 2.5% (1/40). There was a correlation (r = 0.361, p < 0.022) between amphistosome and schistosome infections.

### Logistic regression analysis

Our findings from the predictive logistic regression model indicate that body condition based on the KFI was not influenced by parasite infestation (Table 3). We also observed that parasite burden was not influenced by sex. However, age had an influence on parasite burden because older animals above 15 years were 1.5 times more prone to infection than those aged 1-5 years (odds ratio = 1.5, 95% CI: 1.1-2.4). The Hosmer-Lemeshow goodness-of-fit check showed that the model fitted the data well at ROC = 0.76.

**Table 3:** Results from a predictive logistic regression model for parasitic infection in Kafue lechwe antelopes (n = 40) -October to December, 2005.

Variable	Level	OR	P-Value	95% CI
Body C score	Good	1	-	-
	Fair	0.4	0.53	0.6-1.1
	Poor	0.2	0.19	0.1-1.4

Age category	1-5 yrs	1	-	-
	5-10 yrs	1.2	0.00	
	10-15 yrs	0.9	0.01	0.6-1.2
	>15 yrs	1.5	0.03	1.1-2.4

Sex	Male	1	-	-
	Female	0.34	0.15	0.8-1.3

Assessment of the model fit to the observed data showed insignificant differences between observed and predicted values (HL (χ^2^) (7) = 6.43; p = 0.49), hence indicating its high validity. The reliability of the model was relatively high (ROC = 0.76).

## Discussion

In ecological management, monitoring body condition of wild game is an important tool for monitoring the nutritional conditions of the habitat, and such information can also be used to assess the negative effect of parasites on the host [12]. Although several techniques for estimating body condition in wild ungulates have been developed [26,27], the kidney fat index (KFI) is the most widely used technique because it is regarded as the most reliable indicator of body condition in wild game [26]. Hence, in the present study, we investigated the impact of worm infestations on body condition of the Kafue lechwe by using the KFI as a proxy measure. Stien et al [28] pointed out that timing in carrying out observational studies aimed at detecting the impact of worm infestation on susceptible hosts is crucial because it is important to take into account the lag phase between the time of infection and the maximum parasite intensity in the infected hosts. Phiri et al [5] observed that increase in worm infection in the Kafue basin begins in June/July when the floods recede extending up to November/December when the rain season begins on the Kafue basin. Taking these observations into account, we carried out our investigations between October and December during the peak of the dry season when the parasite intensity is expected to be high in the infected animals. We only managed to evaluate 40 Kafue lechwe antelopes because these wild animals are protected by law because of the endangered nature of the *Kobus leche kafuensis *subspecies [4].

Our findings indicate that there was no statistically significant association between KFI and parasitic burden (Table 3), suggesting that the observed parasitic infections were unlikely to cause adverse body conditions in the infected animals. These findings suggest that at present, parasitic infections might not play a pivotal role to lechwe debilitation and consequently to population reduction on the Kafue basin. This is contrary to observation made by Gallagher et al [9], who reported that worm infestation was estimated to kill about 14% of the Kafue lechwe in the Kafue basin and suggested that worm infections were likely to be the major cause of the population reduction observed in the Kafue lechwe antelopes. However, age was shown to have a significant effect, as animals older than 15 years were one and half times more likely to carry parasites than those aged between 1 and 5 years (odds ratio = 1.5, 95% CI: 1.1-2.4). Sex had no influence on the parasitic burden. Gallagher et al [9] recorded a higher prevalence of fasciolosis in females than males, while Stafford [10] recorded a higher prevalence in the males than the females. The variations obtained from the two studies [9,10] suggest that sex has no influence on parasitic burdens in lechwe and this supports our current findings.

Tick infestation rates of 17.5% (7/40) in our study are lower than those associated with low productivity and loss of weight gain in cattle [29,30]. This could be attributed to the semi aquatic nature of the lechwe, which spends most of its time in water submerged up to shoulder. It is likely that the aquatic tendency might not support the survival of most ticks. Similarly, Stafford [10] recorded a low prevalence of tick infestation on lechwe in the Kafue basin. Apart from ticks, oestrid larvae (*Strobiloestrous vanzyli*) were also observed in lechwe. These parasites are common in lechwe, and their presence has not been linked to mortalities in lechwe [10].

## Conclusion

This study highlights species of parasites present in the Kafue lechwe antelope and provides a broad assessment of body condition relative to parasite loads. Based on these comparisons, parasite load does not appear to have a significant impact on the health and condition of lechwe. Therefore, factors other than parasites should be considered in future investigations designed to evaluate factors most important to maintenance of a viable lechwe population in the Kafue basin.

## Competing interests

The authors declare that they have no competing interests.

## Authors' contributions

MM was involved in the sampling, data collection and analysis and preparation of the manuscript. HMM was involved in the sampling, data collection and preparation of the manuscript. AMN was involved in sampling and data collection. JBM was involved in the sampling, data collection and analysis as well as the preparation of the manuscript. DB was involved in data analysis and manuscript writing. VMS was involved in the sample collection and preparation of the manuscript. All authors participated in the preparation of the manuscript.
